# Inhibitory effects of Schisandrin C on collagen behavior in pulmonary fibrosis

**DOI:** 10.1038/s41598-023-40631-6

**Published:** 2023-08-18

**Authors:** Mingchen Xu, Chenghe Zhao, Haiming Song, Chunmei Wang, He Li, Xudong Qiu, He Jing, Wenyue Zhuang

**Affiliations:** 1 Department of Molecular Biology Test Technique, College of Medical Technology, Beihua University, No. 3999 Binjiang East Road, Fengman District, Jilin, 132013 China; 2 Department of Pharmacology, College of Pharmacy, Beihua University, Jilin, China; 3 Department of Hand Surgery, Affiliated Hospital, Beihua University, Jilin, China

**Keywords:** Cell biology, Diseases

## Abstract

Pulmonary fibrosis (PF) is a serious progressive fibrotic disease that is characterized by excessive accumulation of extracellular matrix (ECM), thus resulting in stiff lung tissues. Lysyl oxidase (LOX) is an enzyme involved in fibrosis by catalyzing collagen cross-linking. Studies found that the ingredients in schisandra ameliorated bleomycin (BLM)-induced PF, but it is unknown whether the anti-PF of schisandra is related to LOX. In this study, we established models of PF including a mouse model stimulated by BLM and a HFL1 cell model induced by transforming growth factor (TGF)-β_1_ to evaluate the inhibition effects of Schisandrin C (Sch C) on PF. We observed that Sch C treatment decreased pulmonary indexes compared to control group. Treatment of Sch C showed a significant reduction in the accumulation of ECM as evidenced by decreased expressions of α-SMA, FN, MMP2, MMP9, TIMP1 and collagen proteins such as Col 1A1, and Col 3A1. In addition, the expression of LOX in the lung tissue of mice after Sch C treatment was effectively decreased compared with the MOD group. The inhibition effects in vitro were consistent with those in vivo. Mechanistic studies revealed that Sch C significantly inhibited TGF-β_1_/Smad2/3 and TNF-α/JNK signaling pathways. In conclusion, our data demonstrated that Sch C significantly ameliorated PF in vivo and vitro, which may play an important role by reducing ECM deposition and inhibiting the production of LOX.

## Introduction

Coronavirus disease 2019 (COVID-19) is an extremely transmissible infectious respiratory disease that has caused a continuing pandemic since early 2020. Survivors of COVID-19 possibly face long-term pulmonary sequelae such as pulmonary fibrosis (PF)^1^. PF is a chronic, progressive and fibrosing interstitial pulmonary disease, characterized by the transformation of fibroblasts into myofibroblasts, recruitment of inflammatory cells and gradual deposition of extracellular matrix (ECM)^2^. These changes harden the lung and break its structure and function leading to shortness of breath and death^3,4^. Many factors, such as respiratory virus infection, environmental, occupational exposure, and so on, can cause PF^5,6^. And idiopathic pulmonary fibrosis (IPF) is a chronic, progressive and irreversible fibrotic disease of unknown etiology^7^. Consequently, there is an urgent need to find the drugs to slow the progression of PF.

ECM accumulation is one of the important pathological processes in the occurrence and development of PF^8^. The configuration of ECM staging components into a complex of fibers and networks requires the secretion, modification, assembly, and stabilization of tropoelastin monomers and collagen fibers^9^. The construction of these ECM constituents imparts the gain of biophysical properties those are needed to support pulmonary physiologic function^10^. Under pathological situations, over- accumulated collagen and overactivated ECM crosslinking promote organ fibrosis. And ECM accumulation is regulated by the balance of the protease and anti-protease, such as matrix metalloproteinases (MMPs) and tissue inhibitor of matrix metalloproteinases (TIMIPs)^11^. Aberrant activation and differentiation of play an important role in the ECM, and transforming growth factor (TGF)-β_1_ is a well-known inducing factor^12,13^. Studies have presented that the expression of lysyl oxidase (LOX) was significantly increased in the lung tissue of PF, and PF can be alleviated via inhibiting LOX expression^14,15^. LOX, a copper-dependent enzyme, is the most pivotal enzyme for ECM crosslinking^16^. LOX is activated when unified with copper in the Golgi and is afterward secreted extracellularly to crosslink collagen and elastin. Regulation of LOX is relative to a series of signaling pathways, including TGF-β_1_/Smad and tumor necrosis factor (TNF)-α/c-Jun N-terminal kinase (JNK) and so on^17^. Activation of TGF-β1 regulates fibrotic proteins and promotes collagen synthesis^18,19^, TNF-α can induce inflammation and JNK activation, which further leads to cell death^20,21^, so TGF-β1/Smad and TNF-α/JNK regulation may plays a role in PF. Therefore, inhibition of LOX expression has been recognized as a potential treatment strategy of PF^22^.

Schisandrin C (Sch C), one of the lignans of Schisandra Chinese, has been shown to inhibit the TGF-β_1_/Smad2/3 signaling pathway which plays an important role in ECM deposition^4,23^. Thus, we hypothesized that Sch C might play a beneficial role in the diminishment of ECM deposition by inhibiting LOX expression contributing to ameliorating PF.

## Results

### Sch C ameliorates body weight and pulmonary indexes in BLM-stimulated mice

**Figure 1:**
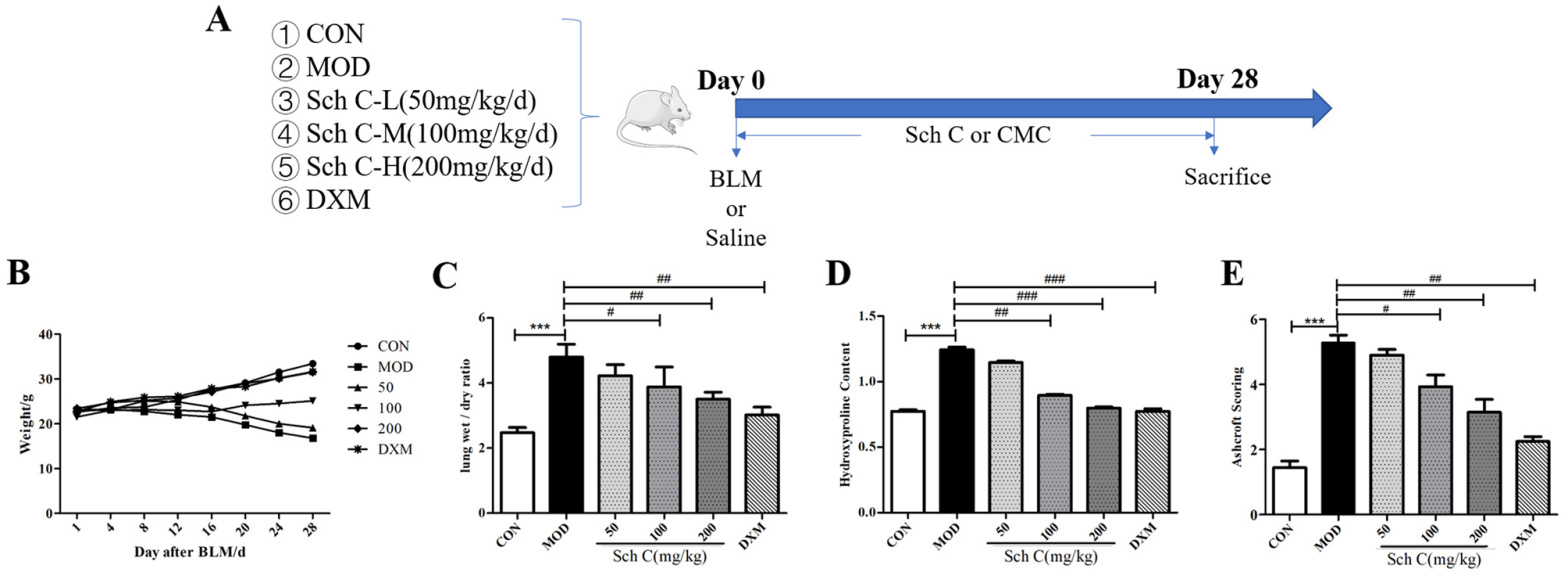
Sch C ameliorated body weight and pulmonary indexes in bleomycin-treated mice. (**A**) Experimental protocol. *CON* Control group, *MOD* BLM group, *Sch C-L* BLM + low dose Sch C group, *Sch C-M* BLM + medium dose Sch C group, *Sch C-H* BLM + high dose Sch C group, *DXM* BLM + DXM group, *CMC* Sodium Carboxymethyl Cellulose. (**B**) Mice body weights were measured in Day 28. (**C**) Wet/dry ratio of lung tissue. (**D**) Total lung tissue hydroxyproline content was measured using assay kit. (**E**) Fibrosis was determined based on Ashcroft scores. Data were presented as the means ± SD (n ≥ 3). *P < 0.05, **P < 0.01, ***P < 0.001 vs CON group; ^*#*^*P* < 0.05, ^*##*^*P* < 0.01, ^*###*^*P* < 0.001 vs MOD.

To examine the protective effectiveness of Sch C on PF, bleomycin (BLM)-induced ICR mice were treated with Sch C, the experimental procedure is revealed in Fig. 1A. In comparison with the CON group, the weight of mice in the MOD group was significantly decreased, which was significantly overturned after Sch C-M and Sch C-H treatment (Fig. 1B). In contrast, the wet/dry ratios of lung tissue was higher in the MOD group than that in the CON group, showing progressive growth in lung tissue edema after BLM-inducement. The wet/dry ratios in the Sch C-M and Sch C-H treated groups were improved in a dose-dependent manner, indicating that pulmonary edema can be ameliorated after Sch C management (Fig. 1C). Further, the content of hydroxyproline (HYP) was decreased in the mice lung tissues in Sch C-M and Sch C-H groups compared to the BLM-induced group (Fig. 1D). Ashcroft fibrotic scores also exposed that the fibrosis score of Sch C treatment groups was much lower than that of the MOD group (Fig. 1E).

### Sch C attenuates pathologic changes in BLM-stimulated mice

**Figure 2:**
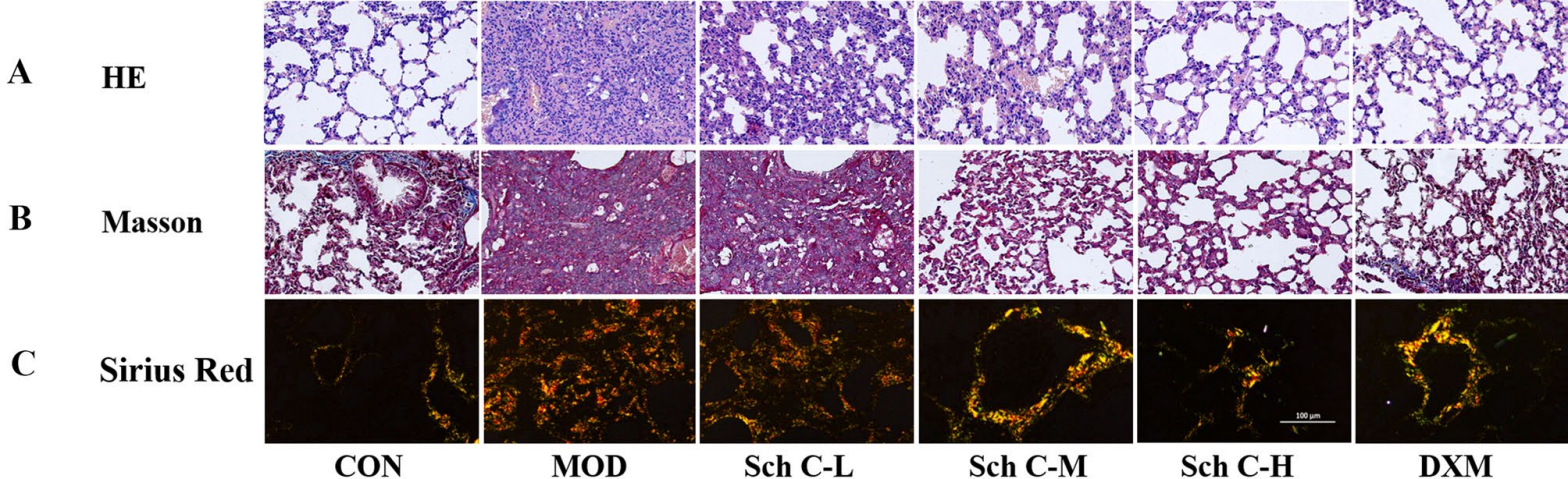
Sch C attenuates pathologic changes in BLM-induced mice. (**A**) Histopathological characteristics of mouse lung tissue sections were used for staining with hematoxylin–eosin (HE, ×200 magnification). (**B**) Collagen fibers (Blue color) in mouse lung tissue sections were visualized by Masson’s staining (×200 magnification). (**C**) Collagen type I (Orange or red color) and Collagen type III (Green color) in mouse lung tissue sections were showed by Sirius Red staining (×200 magnification).

As shown in Fig. 2A, staining of pathological sections discovered histopathological changes in the pulmonary tissues among different treatments. Hematoxylin–eosin (H&E) staining exposed that in the MOD group, the alveolar septum was thickened, the alveolar structure was demolished, and a mass of inflammatory cells penetrated the alveolar and pulmonary interstitium. Sch C-M and Sch C-H intervention significantly prohibited the alveolar septum from thickening and inhibited inflammatory infiltration.

Masson staining showed that compared with the CON group, noticeable features of PF such as deposits palpable collagen, and a mass of blue collagen fibers tangled in the pulmonary interstitium were observed in the MOD group whereas the PF characteristics of the Sch C-M and Sch C-H groups were improved compared with the MOD group (Fig. 2B).

Sirius Red was used to evaluated collagen content in lung tissues and indicated that BLM exposure significantly increased collagen levels in mice, and improvement effects could be detected in Sch C treatment groups (Fig. 2C).

### Sch C inhibits fibroblast-to-myofibroblast differentiation in BLM-stimulated mice

**Figure 3:**
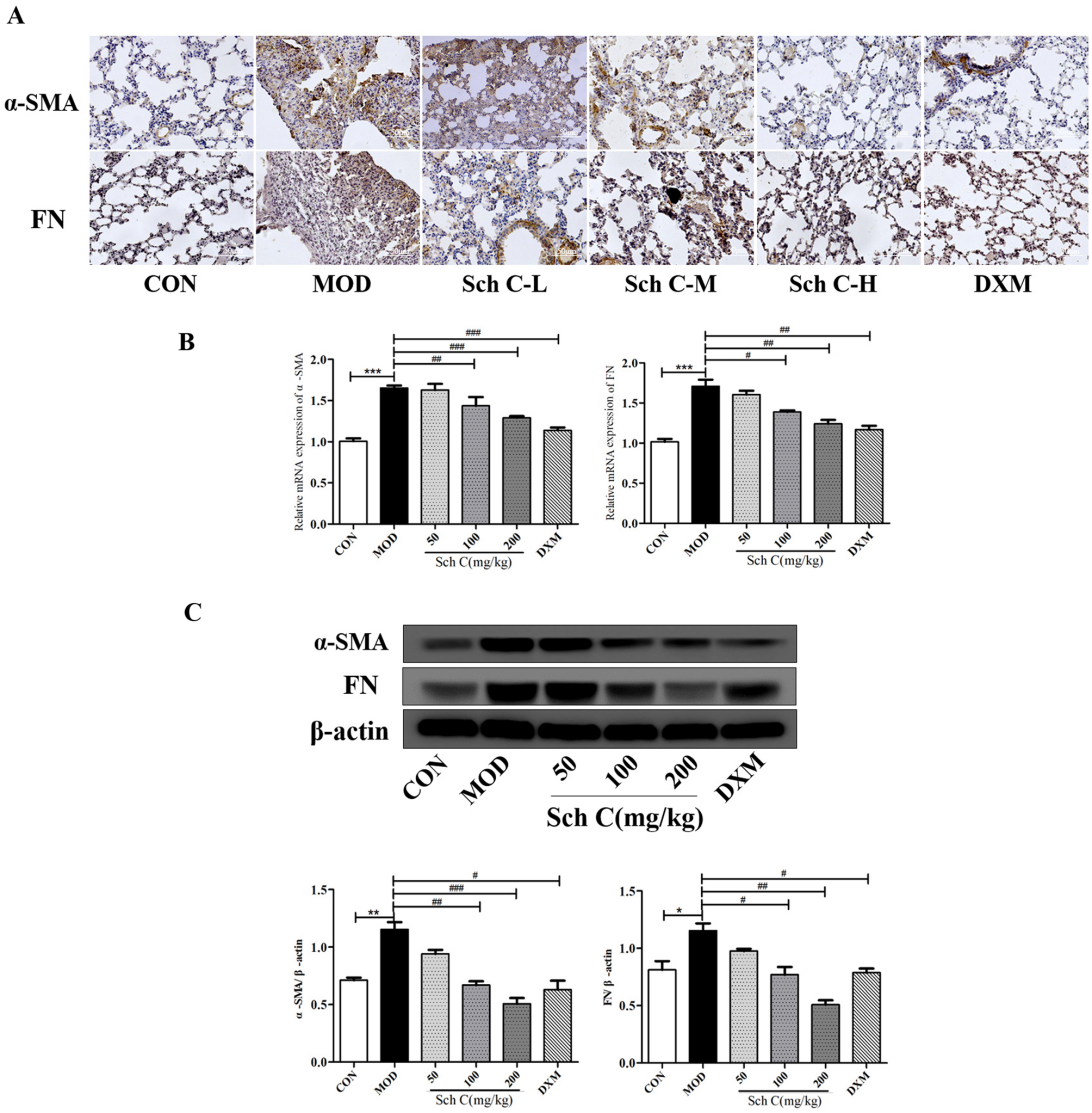
Sch C inhibits fibroblast-to-myofibroblast differentiation in BLM-stimulated mice. Myofibroblasts markers α-SMA and FN in fibroblasts measured using Immunohistochemical staining (**A**, ×200 magnification), qPCR (**B**) and western blot assay. (All blots were clipped according to molecular weight criteria before incubation with primary antibodies). (**C**) The data were normalized to the intensity of β-actin and were expressed relative to the value of the CON group. Data were presented as the means ± SD (n ≥ 3). *P < 0.05, **P < 0.01, ***P < 0.001 vs CON group; ^*#*^*P* < 0.05, ^*##*^*P* < 0.01, ^*###*^*P* < 0.001 vs MOD.

We investigated whether Sch C retarded PF by suppressing the differentiation of fibroblasts to myofibroblasts. Immunohistochemical staining showed that the expressions of alpha-smooth muscle actin (α-SMA) and fibronectin (FN) were significantly increased in pulmonary fibroblasts in the MOD group compared to the CON group. In contrast, after Sch C-M and Sch C-H intervention, the expressions of α-SMA and FN were decreased compared to the MOD group. To evaluate myofibroblast accumulation, lung sections were immunostained with antibodies against α-SMA and FN. The expressions of α-SMA and FN inhibited in the lung parenchyma after Sch C-M and Sch C-H intervention compared with MOD group (Fig. 3A). We also detected α-SMA and FN through qPCR and western blot analysis. The results showed that the mRNA and protein levels of α-SMA and FN were increased in the MOD group compared with the CON group, while they were inhibited after Sch C-M and Sch C-H treatment (Fig. 3B,C). These results demonstrated that Sch C intervention effectually inhibited the differentiation of fibroblast to myofibroblasts.

### Sch C inhibits ECM accumulation in BLM-stimulated mice

**Figure 4:**
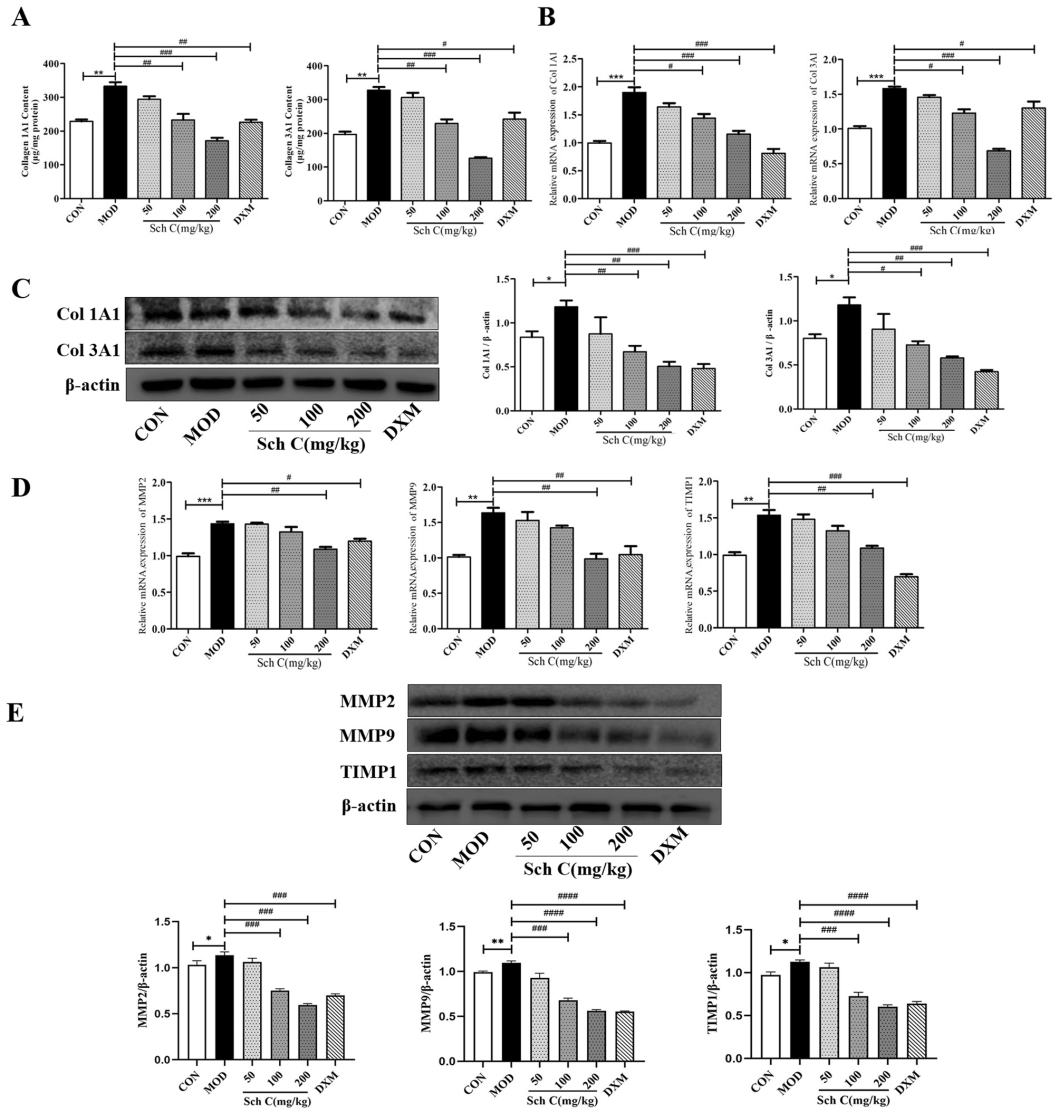
Sch C inhibited ECM accumulation in BLM-stimulated mice. (**A**) Contents of Col 1A1 and Col 3A1. (**B**) Relative expressions of Col 1A1 and Col 3A1 mRNAs. (**C**) Relative expressions of Col 1A1 and Col 3A1 proteins. (**D**) Relative expressions of MMP2, MMP9, and TIMP1 mRNA. (**E**) Relative expressions of MMP2, MMP9 and TIMP1 proteins. The data were normalized to the intensity of β-actin and are expressed relative to the value of the CON group. Data were presented as the means ± SD (n ≥ 3). *P < 0.05, **P < 0.01, ***P < 0.001 vs CON group; ^*#*^*P* < 0.05, ^*##*^*P* < 0.01, ^*###*^*P* < 0.001 , ^*####*^*P* < 0.0001 vs MOD.

Since the accretion of ECM proteins is a link with PF, we detected the effects of Sch C on the expressions of collagen 1A1 (Col 1A1), Col 3A1, MMPs and TIMIPs in BLM-mediated mice. Col 1A1 and Col 3A1 levels in lung tissues were detected using ELISA. In the BLM group, Col 1A1 and Col 3A1 levels were significantly up-regulated compared with the CON group. After Sch C-M and Sch C-H intervention, the expression of Col 1A1 and Col 3A1 were significantly inhibited in comparison with the BLM group (Fig. 4A). QPCR and western blot results were consistent with those of ELISA (Fig. 4B,C). The results showed that mRNA and protein expression levels of matrix metalloproteinases-2 (MMP2), matrix metalloproteinases-9 (MMP9), and tissue inhibitor of matrix metalloproteinases (TIMP1) in the MOD group were outstandingly higher than those in the CON group, whereas Sch C-M and Sch C-H treatment significantly reduced the levels of those genes (Fig. 4D,E).

### Sch C inhibits the level of LOX in BLM-stimulated mice

**Figure 5:**
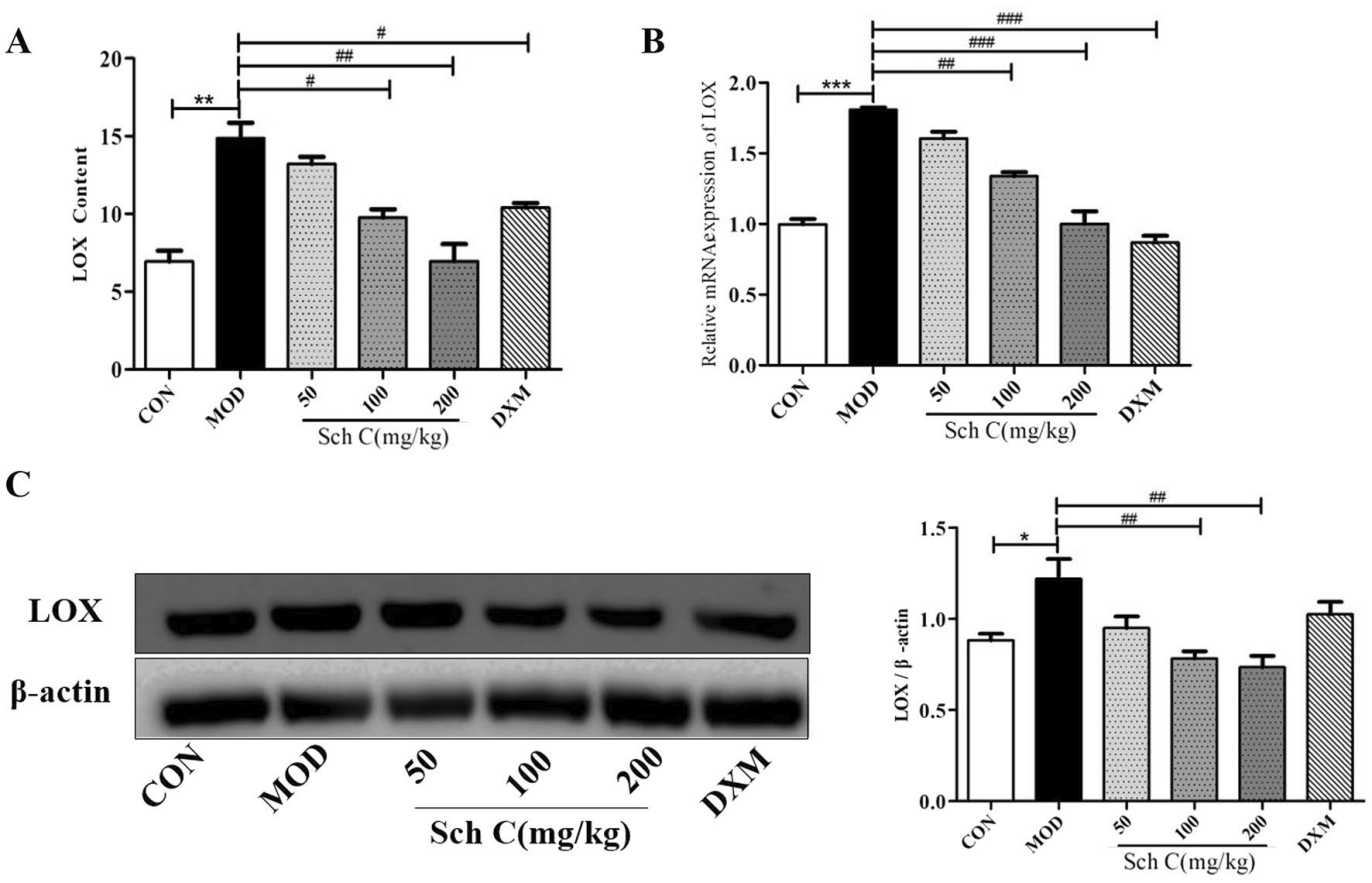
Sch C inhibits the level of LOX in BLM-stimulated mice. (**A**) Content of LOX. (**B**) Relative expression of LOX mRNA. (**C**) Relative expression of LOX protein. The data were normalized to the intensity of β-actin and are expressed relative to the value of the CON group. Data were presented as the means ± SD (n ≥ 3). *P < 0.05, **P < 0.01, ***P < 0.001 vs CON group; ^*#*^*P* < 0.05, ^*##*^*P* < 0.01, ^*###*^*P* < 0.001 vs MOD.

Collagen biosynthesis and intermolecular crosslinking are complicated biological processes, in which LOX plays an important role^22^. Therefore, the level of LOX in lung tissues was analyzed. In Fig. 5A, the content of LOX in the MOD group has significantly exposed an increase compared with the CON group. After Sch C-M and Sch C-H intervention, the expression of LOX was significantly reduced compared with the MOD group, which was suggestive of the inhibition of collagen crosslinking (Fig. 5B,C).

### Sch C down-regulates TGF-β_1_/Smad2/3 and TNF-α/JNK signaling pathways in BLM-stimulated mice

**Figure 6:**
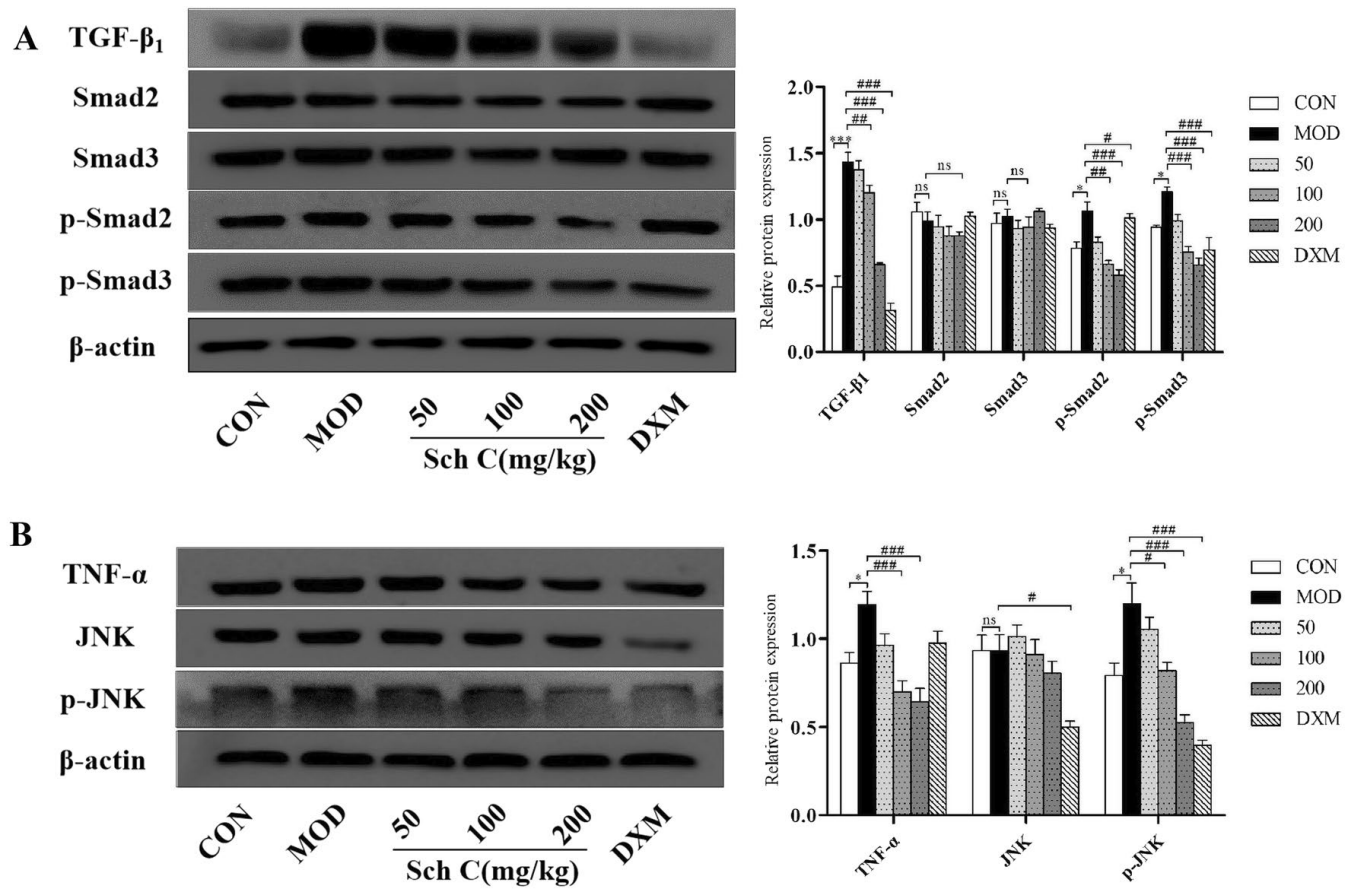
Sch C down regulated the TGF-β_1_/Smad2/3 and TNF-α/JNK signaling pathways in BLM-stimulated mice. (**A**) Western blot bands of TGF-β_1_/Smad2/3 signaling pathways proteins in lung tissue of mice. (**B**) Western blot bands of TNF-α/JNK signaling pathways proteins in lung tissue of mice. The data were normalized to the intensity of β-actin. Data were presented as the means ± SD (n ≥ 3). *P < 0.05, **P < 0.01, ***P < 0.001 vs CON group; ^*#*^*P* < 0.05, ^*##*^*P* < 0.01, ^*###*^*P* < 0.001 vs MOD, ns, non-significant.

To further explore the underlying mechanism, we examined the effect of Sch C on the TGF-β_1_/Smad2/3 and TNF-α/JNK pathways in BLM-induced mice. The result showed that TGF-β_1/_Smad2/3 pathway was activated in the MOD group. Western blot analysis showed that protein expressions of TGF-β_1_ and p-Smad2/3 in pulmonary tissues in the MOD group were effectively increased, and were down-regulated by Sch C treatment. Whereas, the protein levels of total Smad2/3 remained unchanged. The results demonstrated that Sch C-M and Sch C-H treatment attenuates PF by suppressing the TGF-β_1/_Smad2/3 signaling pathway (Fig. 6A).

The expression levels of TNF-α and p-JNK in the MOD group were significantly increased. However, total JNK protein levels did not change. The results proved that Sch C-M and Sch C-H intervention inhibited the TNF-α/JNK pathway in BLM-induced mice (Fig. 6B).

### Sch C inhibits the differentiation and migration of TGF-β_1_-stimulated HFL1 cells

**Figure 7:**
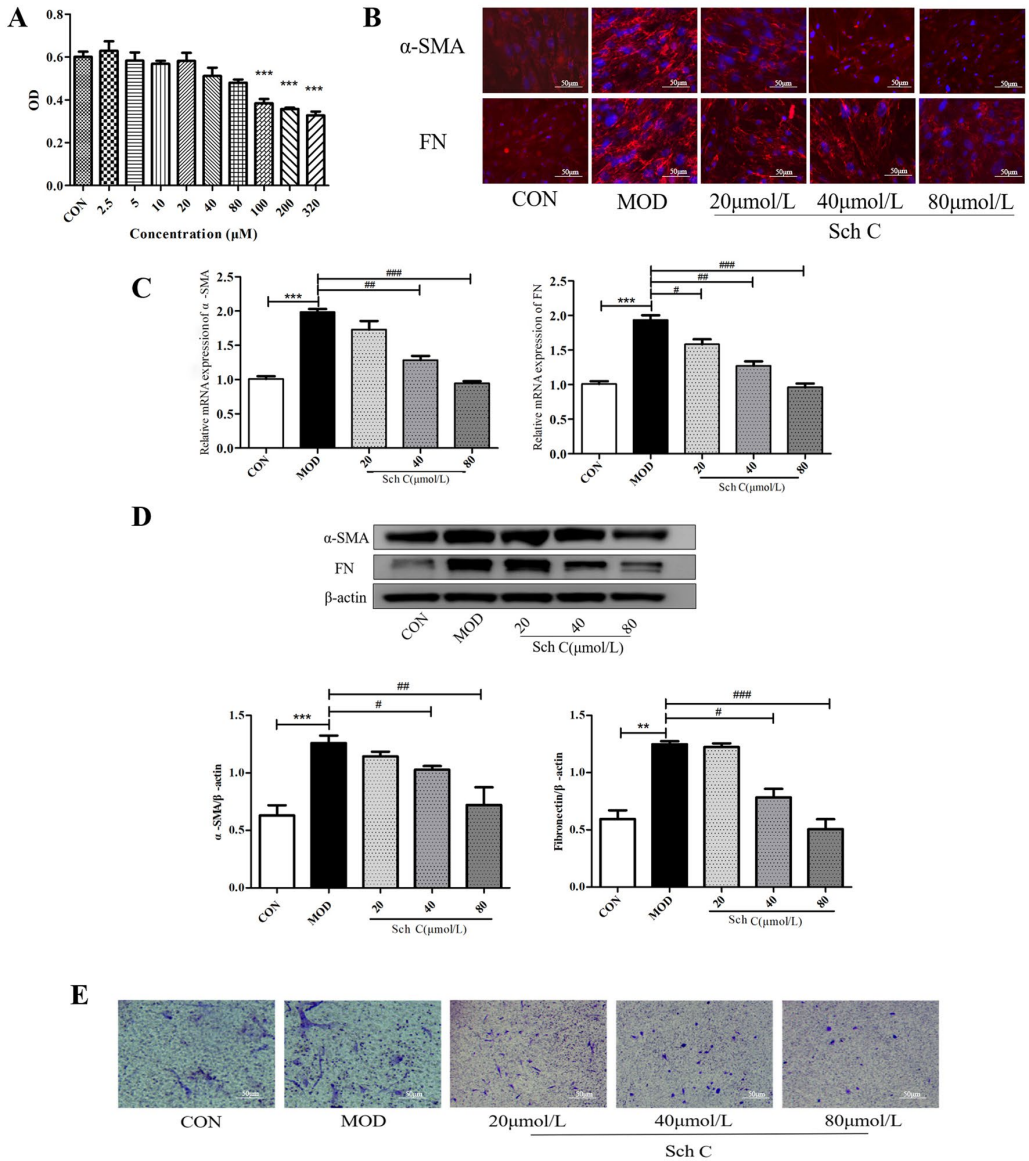
Sch C inhibited the differentiation and migration of TGF-β_1_-stimulated HFL1 cells. (**A**) Viability of cells cultured with different concentrations of Sch C by MTT assay. (**B**) Levels of α-SMA and FN in HFL1cells were measured using immunofluorescence (×200 magnification). (**C**,**D**) QPCR and western blot were used to measure mRNA and protein levels of α-SMA and FN. (**E**) Fibroblast migration ability determined by Transwell assay (×200 magnification). The data were normalized to the intensity of β-actin and are expressed relative to the value of the CON group. Data were presented as the means ± SD (n ≥ 3). *P < 0.05, **P < 0.01, ***P < 0.001 vs CON group; ^*#*^*P* < 0.05, ^*##*^*P* < 0.01, ^*###*^*P* < 0.001 vs MOD.

As Sch C could decrease the expression of α-SMA and FN in BLM-stimulated PF, we predicted the anti-fibrotic effects of Sch C in lung fibroblasts. Therefore, we evaluated the inhibition of Sch C on fibroblasts differentiation induced by TGF-β_1_. First, MTT assay was used to measure the effect of Sch C on HFL1 cell viability. We selected 3 concentrations (20 μM, 40 μM, 80 μM) which didn’t affect the cell viability for subsequent experiments (Fig. 7A). Immunofluorescence staining showed that TGF-β_1_-inducement increased the expressions of α-SMA and FN which were decreased by Sch C-M and Sch C-H treatment (Fig. 7B). Consistently, the concentration-dependent inhibitory effects of Sch C on α-SMA and FN expression levels were also detected in qPCR and western blot assays (Fig. 7C,D). Furthermore, we inspected the migration of fibroblasts by Transwell assay. As shown in Fig. 7E, Sch C-M and Sch C-H treatment significantly suppressed the cellular migration of TGF-β_1_-stimulated HFL1.

### Sch C inhibits the expressions of MMP2, MMP9, TIMP1, Col 1A1 and Col 3A1 in TGF-β_1_-stimulated HFL1 cells

**Figure 8:**
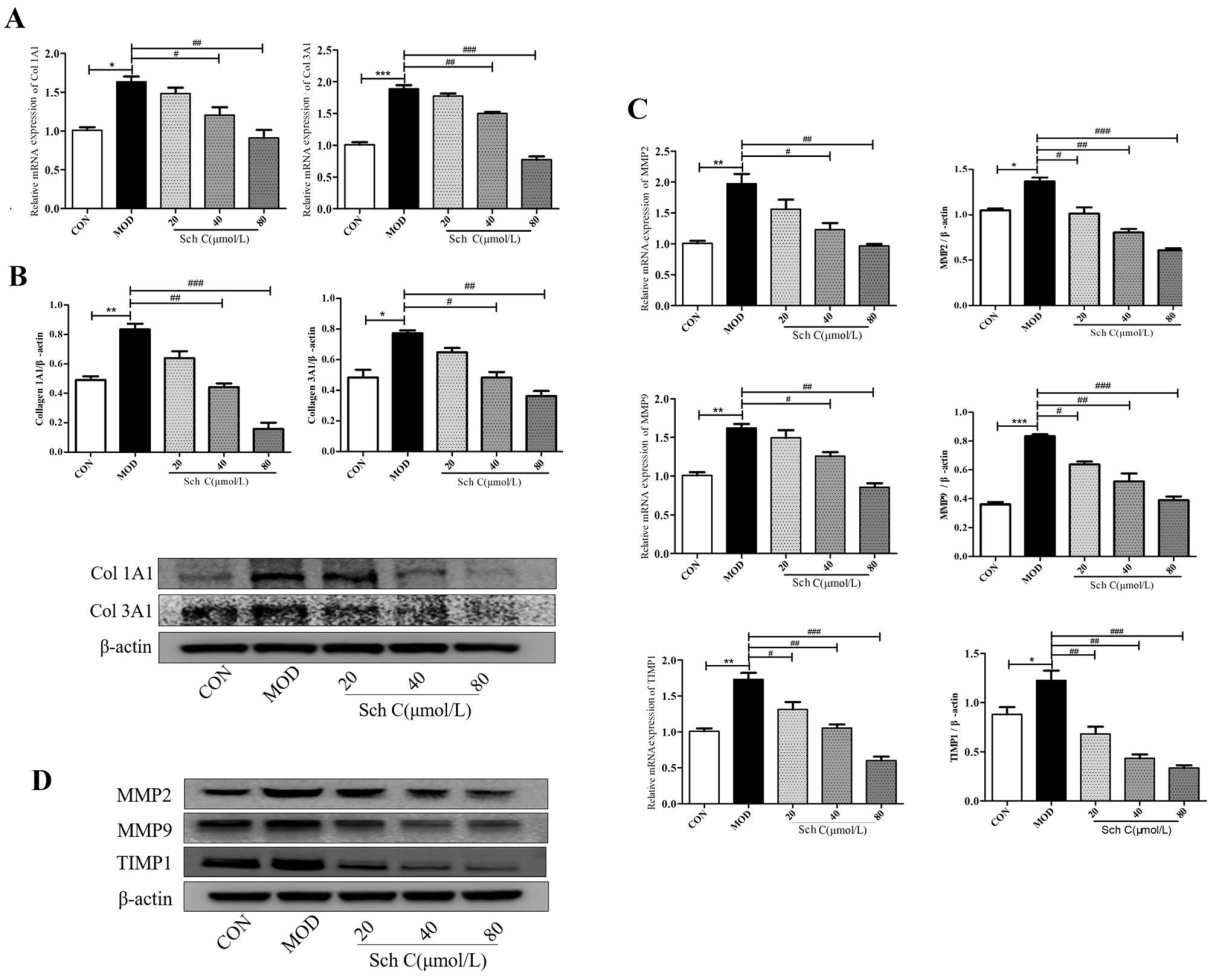
Sch C inhibited the expression of MMP2, MMP9, TIMP1, Col 1A1 and Col 3A1 in TGF-β_1_-stimulated HFL1. (**A**) Relative expression of Col 1A1 and Col 3A1 mRNAs. (**B**) Relative expression of Col 1A1 and Col 3A1 proteins. (**C**) Relative expression of MMP2, MMP9, and TIMP1 mRNAs. (**D**) Relative expression of MMP2, MMP9, and TIMP1 proteins. The data were normalized to the intensity of β-actin and are expressed relative to the value of the CON group. Data were presented as the means ± SD (n ≥ 3). *P < 0.05, **P < 0.01, ***P < 0.001 vs CON group; ^*#*^*P* < 0.05, ^*##*^*P* < 0.01, ^*###*^*P* < 0.001 vs MOD.

The mRNA and protein expressions of Col 1A1 and Col 3A1 were strongly increased in the TGF-β_1_-induced group compared with the CON group. Whereas, Sch C-M and Sch C-H treatment could reverse those alterations (Fig. 8A,B). In addition, MMP2, MMP9, and TIMP1 expression levels showed an obviously increase in the TGF-β_1_-stimulated group compared to the CON group. However, the expressions of MMP2, MMP9, and TIMP1 were inhibited by Sch C (Fig. 8C,D).

### Sch C suppresses the expression of LOX in TGF-β_1_-stimulated HFL1 cells

**Figure 9:**
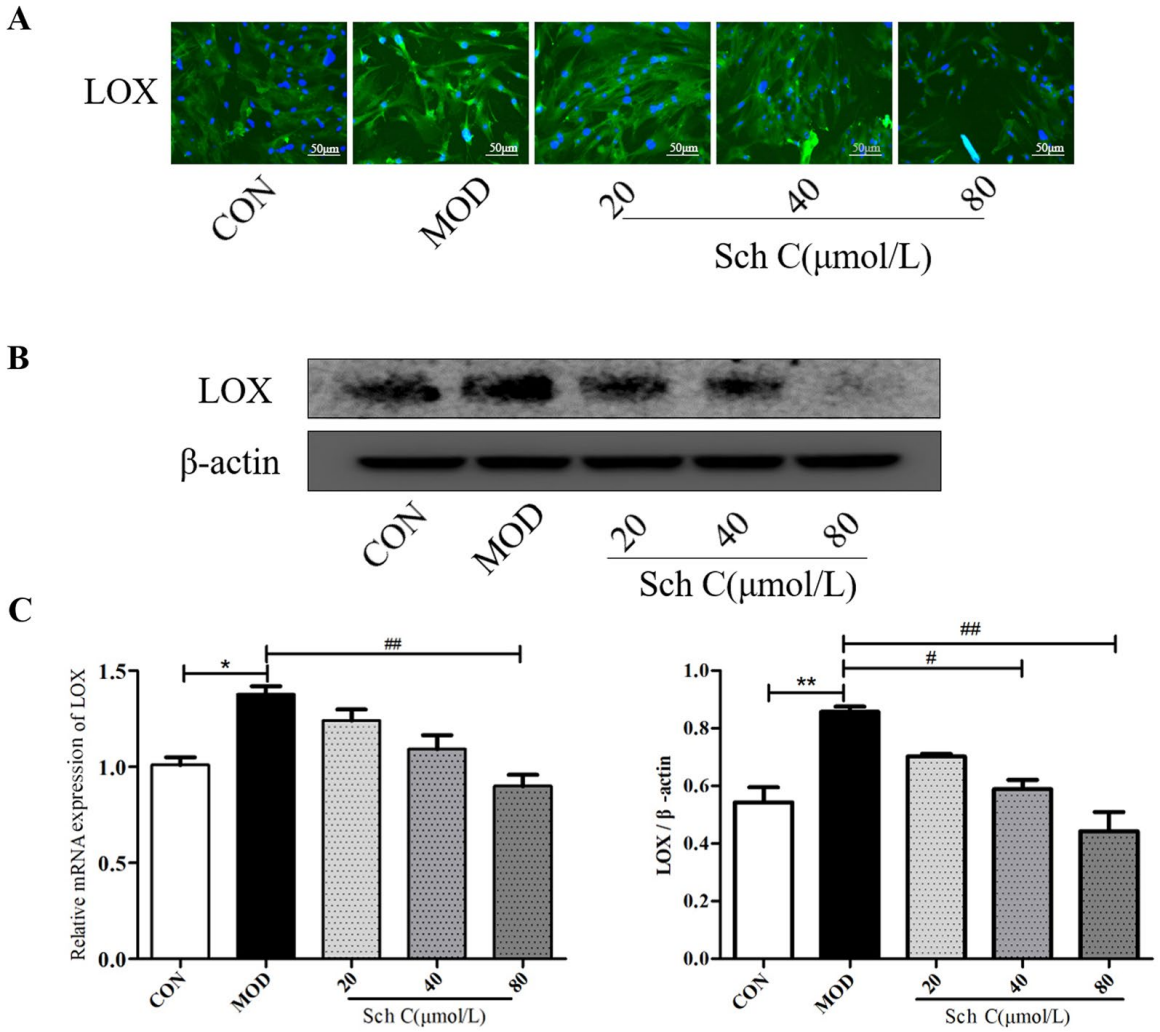
Sch C suppressed the expression of LOX in TGF-β_1_-stimulated HFL1 cells. (**A**) Levels of LOX in HFL1 cells were measured using immunofluorescence (×200 magnification). (B, C) QPCR and western blot were used to measure mRNA and protein levels of LOX. Data were presented as the means ± SD (n ≥ 3). *P < 0.05, **P < 0.01 vs CON group; ^*#*^*P* < 0.05, ^*##*^*P* < 0.01, ^*###*^*P* < 0.001 vs MOD.

After inducement with TGF-β_1_, immunofluorescence staining showed that the expression of LOX was significantly up-regulated in HFL1 cells. Nevertheless, in the presence of Sch C, TGF-β_1_-induced up-regulation of LOX level was reverted (Fig. 9A). The inhibitory effect of Sch C on LOX mRNA and protein expressions was consistent with immunofluorescence staining. We found that the expression of LOX was significantly reduced by Sch C treatment, and the inhibition of JNK signaling pathway by Sch C had a similar result to SP600125 (JNK inhibitor) intervention (Fig. 9B,C).

### Sch C modulates TNF-α/JNK signaling pathway in TGF-β_1_-stimulated HFL1 cells

**Figure 10:**
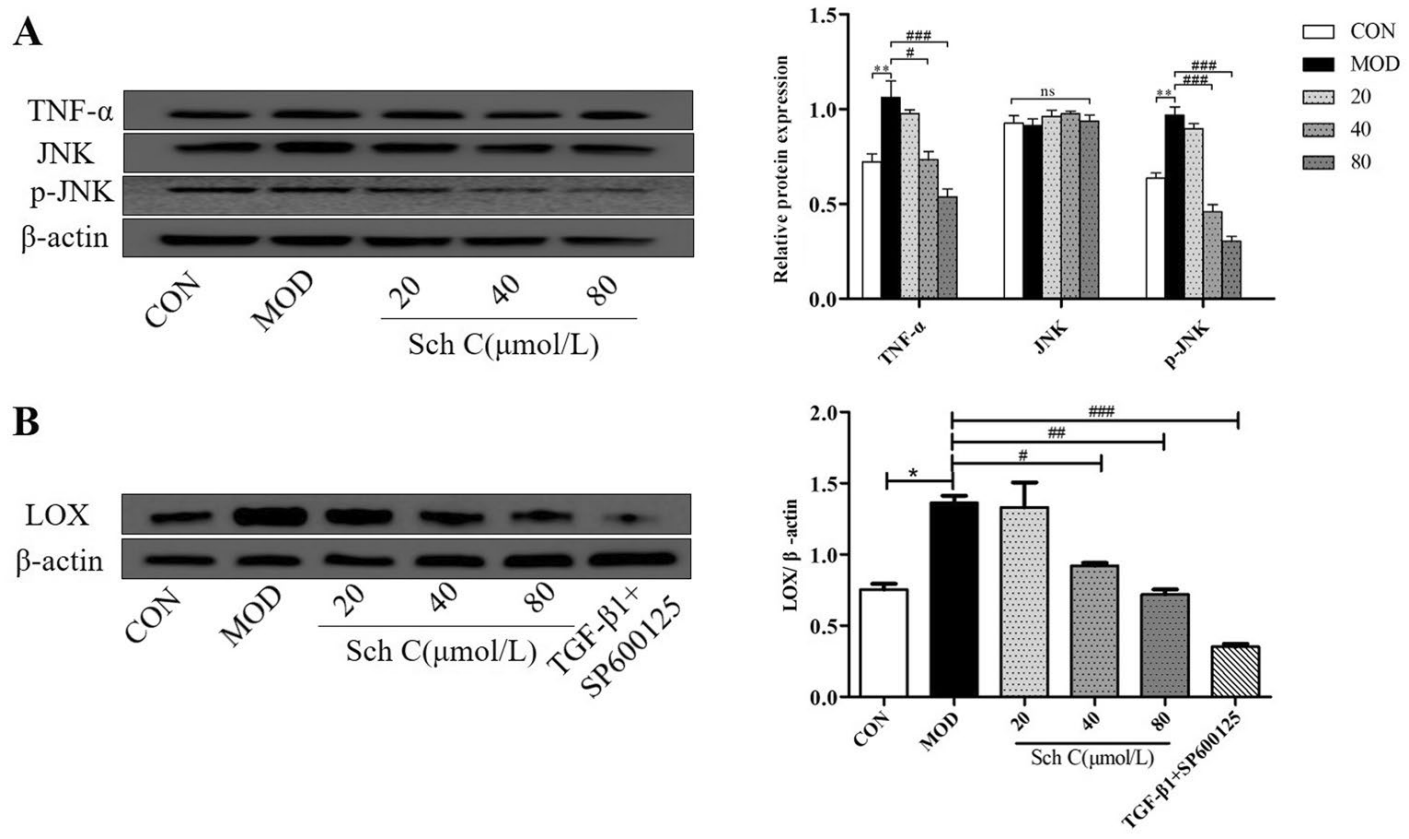
Sch C modulated TNF-α/JNK signaling pathway in TGF-β1-stimulated HFL1 cells. (**A**) The protein expression of TNF-α, JNK and p-JNK was measured by Western blot. (**B**) The protein expression of LOX was measured by Western blot. The data were normalized to the intensity of β-actin and are expressed relative to the value of the CON group. The data were normalized to the intensity of β-actin. Data were presented as the means ± SD (n ≥ 3). *P < 0.05, **P < 0.01 vs CON group; ^*#*^*P* < 0.05, ^*##*^*P* < 0.01, ^*###*^*P* < 0.001 vs MOD.

To measure the mechanism of Sch C in TGF-β_1_-stimulated HFL1 cells, we used western blot to investigate TNF-α/JNK pathway. Our data showed that the expression of TNF-α and p-JNK were significantly higher in answer to TGF-β_1_ stimulation, while TNF-α and p-JNK levels were reduced concurrently in answer to Sch C-M and Sch C-H intervention (Fig. 10).

## Discussion

PF is a progressive interstitial lung disease characterized by acute early inflammation and evolutional fibrosis which result in pathological lung remodeling and respiratory remodeling and breathing failure^24^. Many studies confirm that PF is associated with ECM^25,26^. During the PF progress, many factors prompt the differentiation of fibroblasts into myofibroblasts in lung tissues, and lead to the high expressions of FN, α-SMA, Col 1A1 and Col 3A1^27,28^. Sch C, extracted from *Schisandra chinensis,* has been documented that it can regulate the TGF-β/Smad signaling pathway^23^.The TGF-β/Smad signaling pathway is one of the most important regulatory pathways in ECM deposition^29^. Thus, we hypothesized that Sch C could be attributed to the anti-fibrosis through inhibition of ECM accumulation.

The lungs went through an acute lung injury and an inflammation stage after intratracheal injection of BLM, and ECM deposition occurred in the fourth week^30,31^. In our study, we observed BLM stimulation induced obvious pulmonary injury as evidenced by the increased content of HYP and pulmonary indexes. H&E and Masson staining results showed that the alveolar tissue of MOD group was destroyed, a large number of inflammatory cells were infiltrated, and a mass of blue collagen fibers were observed. Interestingly, Sch C-M and Sch C-H treatment reversed these abnormalities suggesting an anti-fibrotic effect of Sch C.

The differentiation of fibroblasts into myofibroblasts is one of the pathological causes of excessive ECM accumulation^32^. Our results demonstrated that Sch C significantly inhibited the conversion of fibroblasts to myofibroblasts. ECM is a complicated mixture of proteins including Col 1A1, Col 3A1, elastin and so on^32^. Excessive production of collagen has been shown to cause the imbalance between matrix accumulation and degradation, thus contributing to ECM deposition^33^. As compared to the MOD group, Sch C treatment decreased the expression of Col 1A1 and Col 3A1 as indicated by the results of qPCR and western blot in vivo and in vitro. Furthermore, we observed that Sch C suppressed the expressions of MMP2, MMP9 and TIMP1 production. Studies have shown that MMPs and TIMPs limit fibrosis by decreasing ECM components and astricting excessive accumulation of matrix^34,35^.

LOX, an enzyme that affects matrix cross-linking, can regulate ECM remodeling and thus contribute directly to fibrogenesis^36,37^. We found the level of LOX was significantly down-regulated by Sch C-treatment in both BLM-induced mice and TGF-β_1_-induced HFL1 cells.

**Figure 11:**
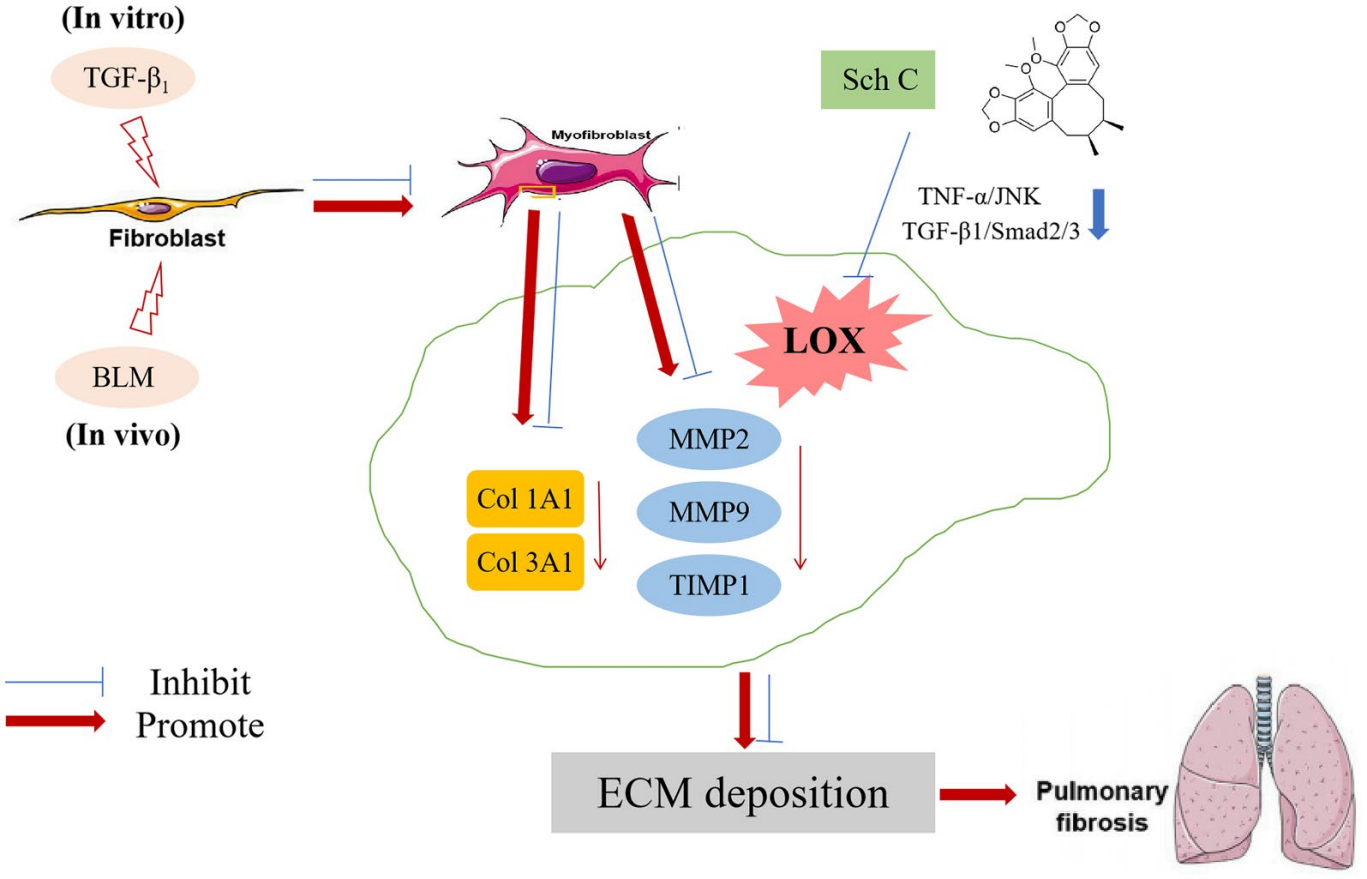
Anti-fibrosis effect of Sch C in vito and in vitro. The machine is mapped using Microsoft PowerPoint 2019 (https://www.microsoft.com/zh-cn/microsoft-365/powerpoint).

TGF-β_1_/Smad2/3 and TNF-α/JNK signaling pathways are upstream pathways of LOX and regulate the activation of LOX^38–41^. Our results indicated that the increased expressions of TGF-β_1_ and smad2/3 phosphorylation after BLM-stimulated was inhibited by Sch C administration in vivo. The interaction between the TNF-α/JNK signaling pathway and the LOX is still not well explored in fibrotic diseases, but our results showed that the inhibition of the JNK pathway was related to the reduction of the expression of LOX. These results suggested that Sch C could inhibit TGF-β_1_/Smad2/3 and TNF-α/JNK signaling pathways, thus reducing the level of LOX (Fig. 11).

## Conclusion

In summary, our study results supplied favorable evidence to prove that Sch C effectively attenuated BLM-stimulated and TGF-β_1_-stimulated PF. Sch C suppressed pathological accumulation of ECM, which may occur by partly inhibiting the TGF-β_1_/Smad2/3 and TNF-α/JNK signaling pathways and decreasing LOX production.

## Materials and methods

### Ethics declarations

All animal procedures were reviewed and approved by the Animal Experimental Ethical Inspection From of Laboratory Animal Center, Beihua University. All methods were performed in accordance with the ARRIVE guidelines 2.0 (https://arriveguidelines.org/arrive-guidelines) as well as relevant guidelines and regulations.

### Drugs and reagents

Sch C, purity: 99.76%, Chengdu Pufei De Biotech Co., Ltd., Chengdu, China; BLM, Nippon Kayaku Co., Ltd. Japan; JNK inhibitor (SP600125), Selleck China Inc. (Shanghai, China); TGF-β_1_, Sigma-Aldrich Inc. (Shanghai, China); The primary anti bodies are both from Abclonal: anti-α-SMA, anti-FN, anti-Collagen type I (Col 1A1), anti-Collagen type III (Col 3A1), anti-LOX, anti-TGF-β_1_, anti-Smad2, anti-Smad3, anti-phosphorylation-Smad2 (p-Smad2), anti-phosphorylation-Smad3 (p-Smad3), anti-JNK, anti-phosphorylation-JNK (p-JNK), anti-MMP2, anti-MMP9, anti-TIMP1, and anti-β-actin. The dilution ratio of all antibodies was 1:1000.

### Animals

Adult male ICR mice (22–25 g; n = 60) were supplied by Changchun Yisi Experimental Animal Co. (Changchun, China), Ltd. The animals were housed in a controlled environment (temperature: 20 ± 2 °C; humidity: 60%; 12 h light/12 h dark cycle). The mice were provided with independent access to food and water and were allowed to adjust to the new environment for 1 week before experimentation. All experimental studies were approved by the Beihua University Committee on Ethics in the Care and Use of Laboratory Animals (YUCE No. 2021-09-09). The experimental protocols were all in accordance with current Chinese legislation and policy.

The mouse model of BLM-induced PF was based on preceding literature published by our team^42^. A total of 60 mice were randomly divided into six groups (10 mice in each group): control group (CON), BLM group (MOD), BLM + low dose Sch C (50 mg/kg, Sch C-L) group, BLM + medium dose Sch C (100 mg/kg, Sch C-M) group, BLM + high dose Sch C (200 mg/kg, Sch C-H) group, and BLM + DXM group. All mice were anesthetized with 1% pentobarbital. (1) Mice in the control group were administered sanitary saline on the first day and were then orally administered 0.5% sodium carboxymethylcellulose for the rest of the experimental period; (2) mice in the BLM group were administered BLM on the first day and were then administered normal sodium carboxymethylcellulose for the rest of the experimental period; (3) mice in the DXM group were administered BLM on the first day and were then orally administered DXM (2 mg/kg) daily for 28 days; (4) mice in the Sch C groups were administered BLM on the first day and were then orally administered Sch C daily for 28 days (50 mg/kg, 100 mg/kg, and 200 mg/kg). 28 days later, the mice were anesthetized by the intraperitoneal injection of pentobarbital for mercy killing, and the left lung tissue was taken for the subsequent analyses.

### Tissue analysis and immunohistochemistry

The left lungs were dipped in 10% formalin for 48 h to fixation and underwent dehydration, paraffin embedding, and sectioning. Paraffin sections (4 μm) were used for HE and Masson staining to value the inflammatory infiltrates and the degree of fibrosis in the lung and 5 μm sections were stained with Sirius red for collagen, the Ashcroft scoring system was for determining the level of fibrosis^42^.

Paraffin-embedded sections of lung tissue were evaluated by immunohistochemistry. After dewaxing and rehydration, tissue sections were dropped with 3% H_2_O_2_ to remove endogenous peroxides. The sections were rinsed in PBS and enclosed by 5% BSA. Subsequently, the sections were incubated with primary antibodies (α-SMA, FN; both in 1:200 thinning; all from cell signaling) overnight at 4 °C. Then the sections were incubated with secondary antibody (1:200 thinning) for 1 h at 37 °C. Sections were dropwise added with DAB at room temperature and the brown colors were measured as positive areas. The sections were then dehydrated and observed under microscope.

### ELISA

The level of collagen I and collagen III in supernatants of homogenized lung tissues were evaluated by ELISA-kits (Langdun, Shanghai, China) following the manufacturer protocols.

### Wet/dry weight ratio assay

Following sacrifice, lung tissues in all groups were separated from the surrounding tissues and harvested, followed by removing blood from the surface. Lung samples were weighed and placed in an electrothermal oven at 60 °C to dry for 72 h. Weight the dry lung tissues and calculate the ratio of wet/dry.

### Hydroxyproline assay

Lung hydroxyproline (HYP) content was measured using hydroxyproline assay kit (Nanjing Jiancheng Bioengineering Research Institute, Nanjing, China) according to the manufacturer’s instructions.

### Cell culture

Fibroblast cell lines HFL1 (Human Lung Fibroblast) were purchased from the National Collection of Authenticated Cell Cultures and cultured in Ham’s F-12K medium (Boster Biological Technology Co. Ltd, USA) containing 10% FBS (Sijiqing, Tianhang Biotechnology Co. Ltd, Hangzhou, China), incubated in a 5% CO_2_ at 37 °C.

Cell experiments were divided into 5 groups: control group (Con), TGF-β_1_ group (MOD, 5 μg/mL), TGF-β_1_ + low dose Sch C (20 μmol/L) group, TGF-β_1_ + medium dose Sch C (40 μmol/L) group, and TGF-β_1_ + high dose Sch C (80 μmol/L) group. To establish the cell model, the Mod group and Sch C groups were stimulated with 8 ng/mL TGF-β_1_ for 24 h, and then culture medium was changed to containing low, medium, high concentrations of Sch C for 48 h.

### MTT assay

HFL1 cells were seeded into 96-well plates with 1 × 10^5^/wells and incubated at 37 °C for 24 h. The cells were then added to dissimilar concentrations of Sch C at 37 °C for 48 h. Subsequently, cells were treated with 5 mg/mL MTT (Beijing Dingguo Changsheng Biotechnology Co. Ltd, Beijing, China) for 4 h. Next, removed MTT solution and added 150 μL DMSO to dissolve the formazan product in each well. The optical density (OD) at 490 nm was measured with a light spectrophotometer.

### Immunofluorescence

HFL1 cells were cultured on coverslips as mentioned previously with Sch C or TGF-β_1_. The cells were fixed with 4% paraformaldehyde for 20 min at 37 °C, followed by blocking with 5% normal goat serum. Then the coverslips were incubated with primary antibodies (α-SMA, FN, LOX; both in 1:200 thinning) overnight at 4 °C and subsequently incubated with Alexa Fluor 594-conjugated Goat Anti-Rabbit IgG (1:200). Fluorescence images were observed with a fluorescence microscope.

### Cell migration assay

Cell migration assay was implemented using Transwell inserts with a membrane with a pore size of 8.0 μm (Corning, MA, USA). The cell (5 × 10^4^) was resuspended in the medium containing 1% serum and seeded into the upper chambers. The bottom chambers were filled with the medium containing 10% serum. After being cultured at 37 °C for 24 h, the cells migrated through the membrane filter, followed by staining with 0.1% crystal violet for 20 min and then observed under an inverted microscope.

### Quantitative real-time PCR

Total RNA from lung tissue or cell were extracted with RNA Isolation Mini Kit (Vazyme, Nanjing, China) and reverse transcribed into cDNA following the manufacturer’s instructions of HiScript III 1st Strand cDNA Synthesis Kit (Vazyme, Nanjing, China). Real-time polymerase chain reaction (PCR) was carried out by utilizing AceQ Universal SYBR qPCR Master Mix (Vazyme) using sense and anti-sense primers, including β-actin, α-smooth muscle actin (α-SMA), FN, Col 1A1, Col 3A1, LOX, MMP2, MMP9, and TIMP1. Table 1 shows the primer sequences. The reliability of the results was normalized to β-actin as an internal reference and calculated using 2^−ΔΔCt^.

**Table 1:** Primers for quantitative RT-PCR, M for mouse, H for human.

Gene		Primer sequence (5′–3′)
H β-actin	F	GGCTGTATTCCCCTCCATCG
	R	CCAGTTGGTAACAATGCCATGT
H Col 1A1	F	GCCAAGACGAAGACATCC
	R	GTCATCGCACAACACCTT
H Col 3A1	F	CTACGGCAATCCTGAACTT
	R	GCAACCATCCTCCAGAAC
H Fibronectin	F	CCCCATTCCAGGACACTTCT
	R	ACAACGTCATAGTGGAGGCA
H α-SMA	F	CTTGAGAAGAGTTACGAGTTG
	R	GATGCTGTTGTAGGTGGTT
H LOX	F	ATTTCTTACCCAGCCGACCA
	R	CTGAAGGCCACAAAGCAAGT
H MMP2	F	CAGCCAACTACGATGATGA
	R	GTGCCAAGGTCAATGTCA
H MMP9	F	GGAAGATGCTGCTGTTCA
	R	CCACCTGGTTCAACTCAC
H TIMP1	F	TGTTGCTGTGGCTGATAG
	R	GTATAAGGTGGTCTGGTTGA
M β-actin	F	GTCCCTCACCCTCCCAAAAG
	R	GCTGCCTCAACACCTCAACCC
M Col 1A1	F	GTGGCGGTTATGACTTCA
	R	CTGCGGATGTTCTCAATCT
M Col 3A1	F	CCTTCTACACCTGCTCCT
	R	CCACTCCAGACTTGACATC
M Fibronectin	F	CAGTTCAGAGGAGCATCAG
	R	GGCATTGTCGTTCAGAGT
M α-SMA	F	TGAAGAGCATCCGACACT
	R	GCCTGAATAGCCACATACA
M LOX	F	GGACATCGGACTTCTTACC
	R	CTTCAGCCACTCTCCTCT
M MMP2	F	GATGGCAAGGATGGACTC
	R	GACCGTTGAACAGGAAGG
M MMP9	F	ACTCACACGACATCTTCC
	R	ATGGTCCACCTTGTTCAC
M TIMP1	F	ATCTGGCATCCTCTTGTTG
	R	GTATAAGGTGGTCTCGTTGA

### Western blot

Mice lungs and HFL1 cells were lysed in lysis buffer (including protease inhibitor and phosphatase inhibitor) for 1 h on ice, and then centrifuged at 12,000 r/min for 5 min, collected the supernatants. Total protein concentration gauged by bicinchoninic acid assay (BCA). Proteins were separated by 10% SDS-PAGE and then transferred onto a PVDF membrane. Then, the membrane was blocked at room temperature for 90 min with 5% skimmed milk and subsequently incubated with the primary antibodies overnight at 4 °C and then with secondary anti-rabbit IgG secondary antibodies for 1 h. The membranes were followed by ECL for luminescence generation and Image J software (Wayne Rasband, NIH USA)^43^ was used to break down the band intensities. Western blot results shown in the various figures were samples from a individual mouse.

### Statistical analysis

Statistical analyses were performed using Graphpad Prism (Prism 5, GraphPad Software, Inc., La Jolla, CA, USA). All raw data are expressed as mean ± standard deviation (SD). One-way repeated measures analysis of variance (ANOVA) followed by the post hoc Tukey’s test was used for comparison between more than two groups. A p-value less than 0.05 was considered significantly.

### Supplementary Information

Supplementary Information.

## Data Availability

The authors confirm that the data supporting the findings of this study are available within the article.
