# Supplementary material for: Inhibitory effects of Schisandrin C on collagen behavior in pulmonary fibrosis
Source: Sci Rep. 2023 Aug 18;13:13475. doi: 10.1038/s41598-023-40631-6 (PMC10439186; doi:10.1038/s41598-023-40631-6)

Supplementary figure 1. Western blot original images of figure 3C.All blots were clipped according to molecular weight criteria before incubation with primary antibodies.

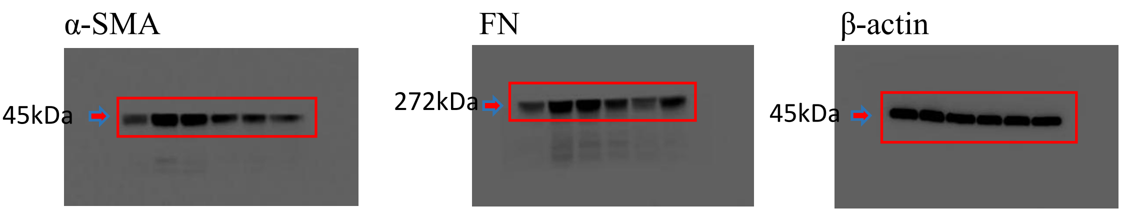

Supplementary figure 2. Western blot original images of Figure4C

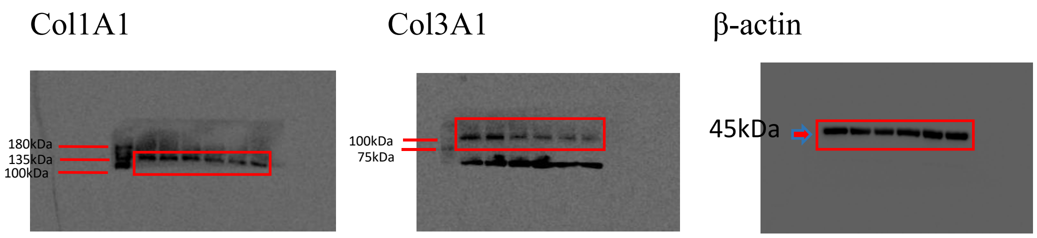

Supplementary figure 3. Western blot original images of Figure4E

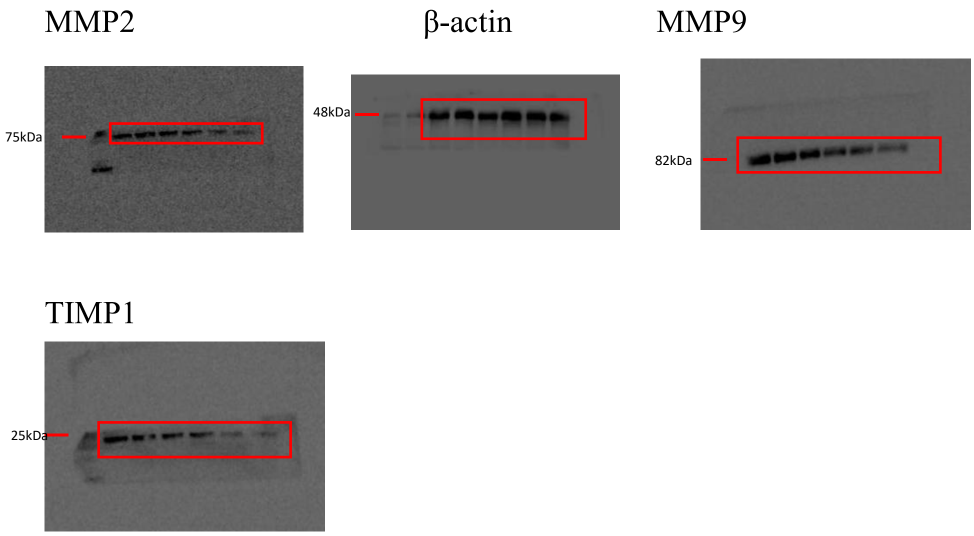

Supplementary figure 4. Western blot original images of Figure5C

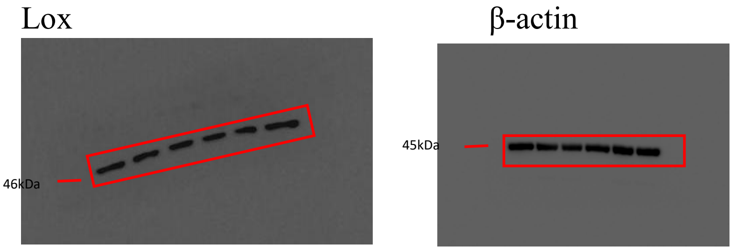

Supplementary figure 5. Western blot original images of Figure6A

TGF-β1

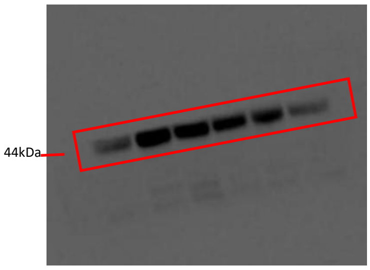

Smad2

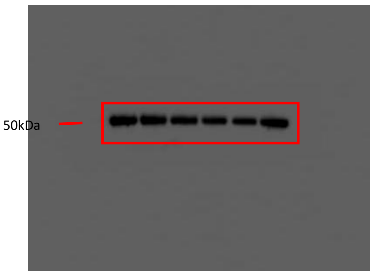

Smad3

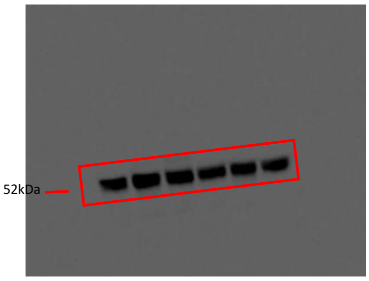

p-Smad2

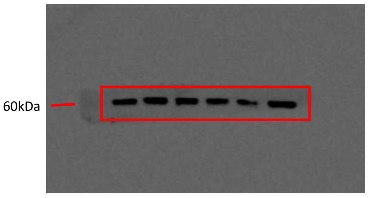

p-Smad3

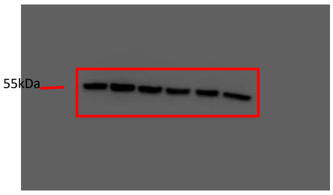

β-actin

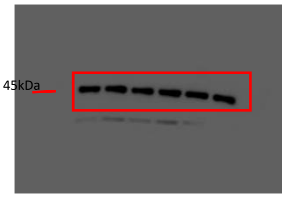

Supplementary figure 6. Western blot original images of Figure6B

TNF-α

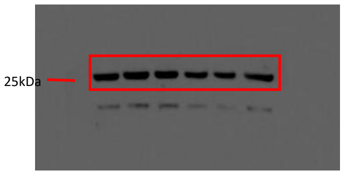

JNK

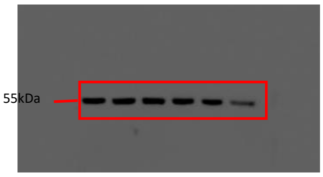

p-JNK

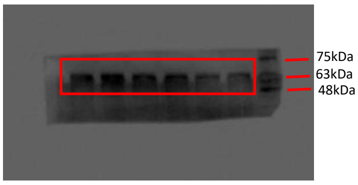

β-actin

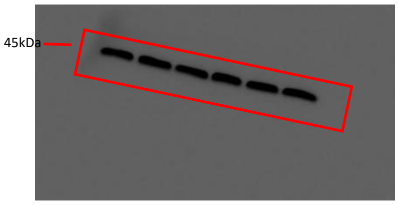

Supplementary figure 7. Western blot original images of Figure7D

α-SMA

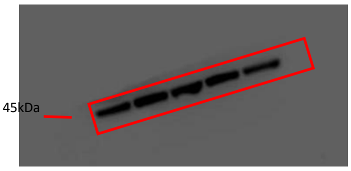

FN

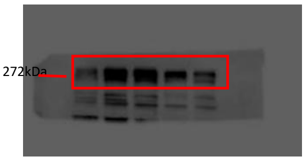

β-actin

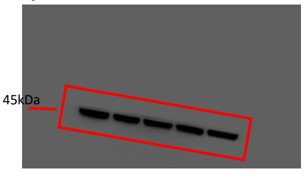

Supplementary figure 8. Western blot original images of Figure8B

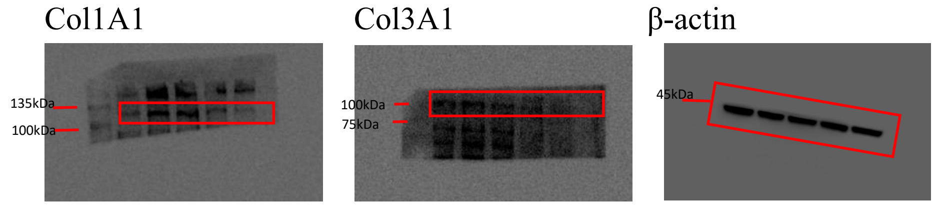

Supplementary figure 9. Western blot original images of Figure8D

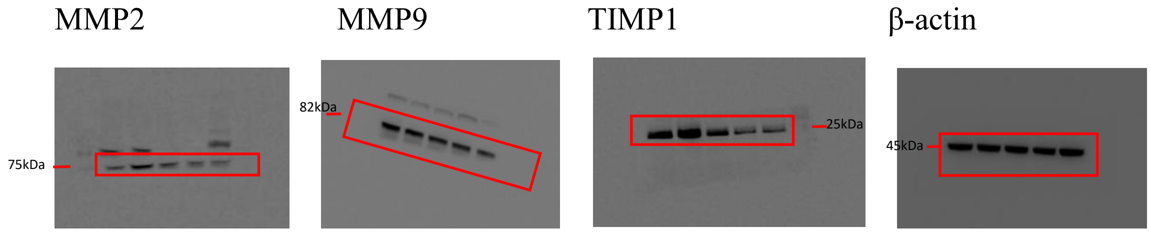

Supplementary figure 10. Western blot original images of Figure9B

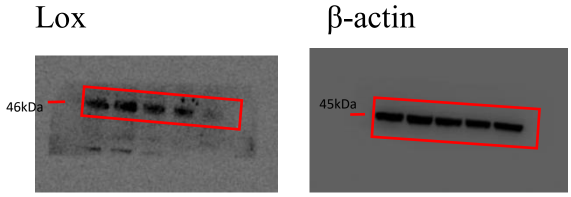

Supplementary figure 11. Western blot original images of Figure10A

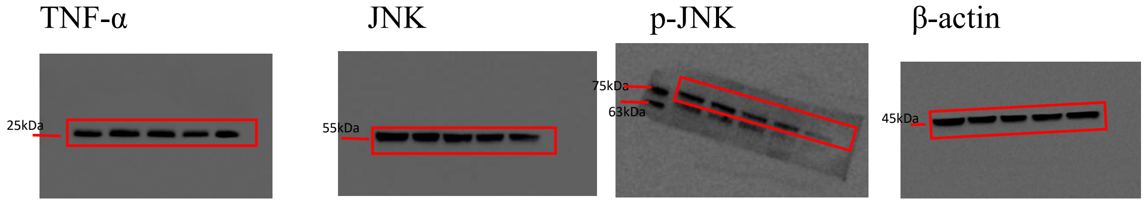

Supplementary figure 12. Western blot original images of Figure10B

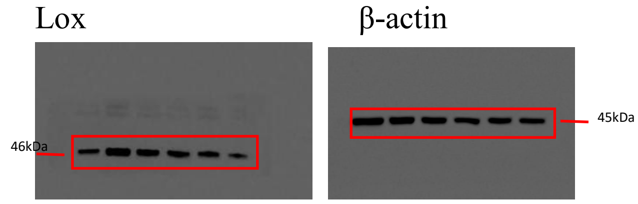

Supplement: Supplementary file 1 — Supplementary Information. [file 41598_2023_40631_MOESM1_ESM.pdf]
